# Treatment of *Leishmania (Leishmania) Amazonensis*-Infected Mice with a Combination of a Palladacycle Complex and Heat-Killed *Propionibacterium acnes* Triggers Protective Cellular Immune Responses

**DOI:** 10.3389/fmicb.2017.00333

**Published:** 2017-03-06

**Authors:** Carolina S. Paladi, Danielle A. M. da Silva, Priscila D. Motta, Daniel M. Garcia, Daniela Teixeira, Ieda M. Longo-Maugéri, Simone Katz, Clara L. Barbiéri

**Affiliations:** ^1^Departamento de Microbiologia, Imunologia e Parasitologia, Escola Paulista de Medicina, Universidade Federal de São PauloSão Paulo, Brazil; ^2^Departamento de Farmacologia, Escola Paulista de Medicina, Universidade Federal de São PauloSão Paulo, Brazil

**Keywords:** cutaneous leishmaniasis, *Leishmania (Leishmania) amazonensis*, palladacycle complex, adjuvant, *Propionibacterium acnes*

## Abstract

Palladacycle complex DPPE 1.2 was previously reported to inhibit the *in vitro* and *in vivo* infection by *Leishmania (Leishmania) amazonensis*. The aim of the present study was to compare the effect of DPPE 1.2, in association with heat-killed *Propionibacterium acnes*, on *L. (L.) amazonensis* infection in two mouse strains, BALB/c and C57BL/6, and to evaluate the immune responses of the treated animals. Foot lesions of *L. (L.) amazonensis*-infected mice were injected with DPPE 1.2 alone, or associated with *P*. *acnes* as an adjuvant. Analysis of T-cell populations in the treated mice and in untreated controls was performed by FACS. Detection of IFN-γ-secreting lymphocytes was carried out by an ELISPOT assay and active TGF-β was measured by means of a double-sandwich ELISA test. The treatment with DPPE 1.2 resulted in a significant reduction of foot lesion sizes and parasite burdens in both mouse strains, and the lowest parasite burden was found in mice treated with DPPE 1.2 plus *P. acnes*. Mice treated with DPPE 1.2 alone displayed a significant increase of TCD4^+^ and TCD8^+^ lymphocytes and IFN-γ secretion which were significantly higher in animals treated with DPPE 1.2 plus *P. acnes*. A significant reduction of active TGF-β was observed in mice treated with DPPE 1.2 alone or associated with *P. acnes*. Moreover, DPPE 1.2 associated to *P. acnes* was non-toxic to treated animals. The destruction of *L. (L.) amazonensis* by DPPE 1.2 was followed by host inflammatory responses which were exacerbated when the palladacycle complex was associated with *P. acnes*.

## Introduction

Parasites belonging to the *Leishmania* genus are etiological agents of cutaneous, mucocutaneous, and visceral diseases in humans and mammals. The World Health Organization estimates that 0.7 to 1.3 million of new cases of cutaneous leishmaniasis and 200,000 to 400,000 of visceral leishmaniasis occur worldwide each year (WHO, 2015). Leishmaniasis comprises one of the diseases included in WHO programs for control and elimination of neglected tropical diseases (Engelman et al., 2016; Molyneux et al., 2016). Among several *Leishmania* species affecting humans, *L. (L.) amazonensis* is one of the causative agents of human cutaneous leishmaniasis in the Amazon region, Brazil, associated with both the simple and diffuse forms of the disease (Lainson and Shaw, 1998).

Pentavalent antimonial compounds are the drugs of choice for the treatment of these diseases, whereas amphotericin B and pentamidine are used as a second-line therapy. However, the use of these compounds is limited by toxicity to the host and development of host resistance to the parasites (Goto and Lindoso, 2010). Miltefosine showed a high efficacy for treatment of visceral leishmaniasis in India and of cutaneous leishmaniasis in Colombia, (Sundar et al., 2002; Soto et al., 2004) but its use is limited by host teratogenicity and development of parasite resistance (Croft and Coombs, 2003). Paromomycin was shown to be effective against cutaneous and visceral leishmaniasis, but its action depends on the causative *Leishmania* species (Thakur et al., 2000). Although oral administration of sitamaquine has shown efficacy in the treatment of visceral leishmaniasis, the drug induces undesirable adverse effects (Jha et al., 2005). Therefore, the development of new leishmanicidal drugs continues to be a priority for the control of leishmaniasis and several compounds including synthetic and natural products extracted from plants and marine sources have exhibited different degrees of efficacy in the treatment of experimental leishmaniasis (Sen and Chatterjee, 2011; Tempone et al., 2011; Coa et al., 2015; Ortiz et al., 2016; Acevedo et al., 2017).

Several of the chemotherapeutic agents against leishmaniasis, targeted to different components of the host immune system, were endowed with immunomodulatory activity (Saha et al., 2011). Recent insights into the host immune responses led to identification of immunomodulators which enhance the efficacy of antileishmanial drugs (Gupta et al., 2011; Seifert et al., 2015). The evidence that antitumor drugs may also display antileishmanial activity has also stimulated the screening of these compounds *in vitro* and in clinical trials (Fuertes et al., 2008; Sanderson et al., 2014). Palladacycle complexes have been pointed out as a new class of antitumoral and antimicrobial agents (Caires, 2007; Elgazwy et al., 2012) and the leishmanicidal and tripanocidal effect of some of these compounds has also been demonstrated (Fricker et al., 2008; Navarro et al., 2008; Matsuo et al., 2010). More recently the activity of the palladacycle complex DPPE 1.2 on *L. (L.) amazonensis* was described (Paladi et al., 2012).

Previous data from our group showed the efficacy of the heat-killed *Propionibacterium acnes* suspension as an adjuvant in murine vaccination with native and recombinant antigens against *Leishmania*, as well as for immunotherapy of canine visceral leishmaniasis (Pinto et al., 2000; Ferreira et al., 2008, 2014). The adjuvant effect of *P. acnes*, in the past classified as *Corynebacterium parvum*, was described due its capability to enhance tumoricidal and phagocytic macrophage function demonstrated in clinical and experimental models (Halpern et al., 1966; Fisher et al., 1990; Salomaa et al., 1995), besides its effects on infection resistance and antibody response (Warr and James, 1975; Brener and Cardoso, 1976). The mechanisms by which *P. acnes* modulates immune response such as increase of proinflammatory cytokine synthesis and modulation of Th2 to Th1 responses were demonstrated in experimental models of tumor and allergy among others (Matsui et al., 1997; Braga et al., 2003; Ananias et al., 2007; Squaiella-Baptistão et al., 2015). The adjuvant effect of the killed *P. acnes* was also demonstrated on vaccination studies against *Trypanosoma cruzi* (Mussalem et al., 2006). All evidence on the important immunomodulatory effects exerted by *P. acnes* and our previous findings on the activity of DPPE 1.2 on *L. (L.) amazonensis* led us to use *P. acnes* associated with this palladacycle complex for the treatment of murine cutaneous leishmaniasis. The murine model is very suitable for studies of *L. (L.) amazonensis* infection, since several mouse strains display different levels of susceptibility to the parasite and mimic the various manifestations of human disease. Within this context, we used the BALB/c strain, highly susceptible to *L. (L.) amazonensis* infection that mimics the anergic form of human diffuse cutaneous leishmaniasis and C57BL/6 that is less susceptible to *L. (L.) amazonensis* infection and presents slow development of lesions (Chang et al., 2003; Scott, 2003; Pereira and Alves, 2008). The data of this study showed that the high efficacy of DPPE 1.2 on *L. (L.) amazonensis*-infected BALB/c and C57BL/6 mice is followed by the modulation of the host immune responses. Furthermore, the leishmanicidal effect of DPPE 1.2, as well as the host inflammatory responses were exacerbated when the pallladacycle complex was associated with *P. acnes*.

## Materials and Methods

### Experimental Animals

Eight-week-old female Golden hamsters were obtained from breeding stocks of Anilab Company, Paulínia (São Paulo, Brazil). Female BALB/c and C57BL/6 mice 6 to 8 weeks old were acquired from Universidade Federal de São Paulo (São Paulo, Brazil). All animals were bred and housed under specific pathogen-free conditions and fed a regular diet. All animal procedures were carried out in strict accordance with the recommendations in the Guide for the Care and Use of Laboratory Animals of the Brazilian National Council of Animal Experimentation^1^. The protocol was approved by the Committee on the Ethics of Animal Experiments of the Institutional Animal Care and Use Committee at the Federal University of São Paulo (Id # CEUA 5332050514).

### Parasites

The *L. (L.) amazonensis* strain used (MHOM/BR/1973/M2269) was kindly provided by Dr. Jeffrey J. Shaw, Instituto Evandro Chagas, Belém, Pará, Brazil and maintained as amastigotes by inoculation into footpads of Golden hamsters every 4 to 6 weeks as previously described (Barbiéri et al., 1990).

### Biphosphinic Palladacycle Complex [Pd(C2, N-S(-)DMPA)(DPPE)]Cl (DPPE 1.2)

The palladacycle compound DPPE 1.2 (Paladi et al., 2012) was obtained from N,N-dimethyl-1-phenylethylamine (DMPA), complexed to 1,2-ethane-bis(diphenylphosphine; DPPE) ligand and synthesized as previously described (Rodrigues et al., 2003). Stock solutions at 1.45 mM were prepared in phosphate buffered saline (PBS; 137 mM NaCl in 10 mM phosphate buffer, pH 7.4) after solubilization in dimethylsulfoxide (final concentration of 0.1%).

### Heat-Killed *P. acnes* Suspension

*Propionibacterium acnes* was obtained from Instituto Adolfo Lutz, São Paulo, S.P., Brazil and cultured in anaerobic medium (Hemobac, Probac, São Paulo, S.P., Brazil) for 3 days at 37°C and washed by centrifugation (Mussalem et al., 2012). The resulting pellets were suspended in 0.9% saline and subjected to continuous water vapor for 20 min at 120°C. The protein concentration of the suspension was determined by the Bradford method (Bradford, 1976).

### *In vivo* Antileishmanial Assays

*In vivo* leishmanicidal activity of either DPPE 1.2 or DPPE 1.2 plus *P. acnes* was evaluated in female BALB/c and C57BL/6 mice 6 to 8 weeks-old infected subcutaneously at the right hind-foot with 1 × 10^5^ *L. (L.) amazonensis* amastigotes freshly prepared as previously described. Fifteen days after infection, the animals were randomly separated in five groups of 12 mice each. Treated animals received in the foot lesions every other day doses of either 60 mg/kg/day (16.8 mg [Sbv]/kg/day) of Glucantime for 1 month (total of 1,200 mg/kg/animal–336 mg [Sbv]/kg/animal) or doses of 320 mg/kg/day of DPPE 1.2 (total of 6.4 mg/kg). Animals treated with DPPE 1.2 plus *P. acnes* received 3 doses of 100 μg of *P. acnes* with an interval of 7 days among them and every other day doses of 320 mg/kg/day of DPPE 1.2 (total of 6.4 mg/kg). Control group received the same number of injections of either PBS or *P. acnes* alone. Infection was monitored once a week by measuring the diameter of foot lesions with a dial caliper (Mitutoyo Corp., Japan). Parasite burden was evaluated by limiting dilution in foot lesions of BALB/c and C57BL/6 mice 15 days after end of the treatment, as previously described (Lima et al., 1997). Treatment of non-infected BALB/c and C57BL/6 mice with either DPPE 1.2 alone or associated with *P. acnes* was also performed.

### Assays for Toxicity

Serum concentrations of urea, creatinine, and transaminases were determined in BALB/c and C57BL/6 mice at the end of treatment, using sets of commercial reagents (Doles Reagentes e Equipamentos para Laboratórios, Ltda, Brazil).

### Evaluation of Immune Responses

The T lymphocyte population was analyzed by fluorescence-activated cell sorter (FACS). The cell suspension from popliteal and inguinal lymph nodes of all BALB/c and C57BL/6 were pooled and counted with Trypan Blue to determine the viability and cellular concentration. After washing with PBS, 1 × 10^6^ lymphocytes were fixed in formalin 1% in PBS for 30 min at 4°C, washed twice in PBS, resuspended in PBS and incubated with monoclonal antibodies either anti-CD3 conjugated to allophycocyanin (APC), or anti-CD4 conjugated to phycoerythrin (PE) or anti-CD8 conjugated to peridinin chlorophyll protein (PerCP; Pharmingen) for 1 h at 4°C, washed twice in PBS, fixed in formalin 1% in PBS for 30 min at 4°C, washed twice in PBS, resuspended in PBS and gated on the basis of forward-angle and right-angle scatter and the fluorescence intensity was analyzed by FACS (FACSCAN – Cell Sorter Becton–Dickinson). The absolute number of each T lymphocyte subpopulation was determined by multiplying the total number of cell suspension obtained from popliteal and inguinal lymph nodes by the percentages of each subset obtained by FACS.

The enzyme-linked immunospot (ELISPOT) assay for detection of IFN-γ-secreting lymphocytes was performed with BD ELISPOT reagents (BD Biosciences). Briefly, the plates were prepared by coating the wells of 96-well PVDF membrane plates with a solution of sterile PBS containing 100 μl of the anti-mouse IFN-γ MAb 5 μg/ml. After overnight incubation at 4°C, the MAb solution was removed by sterile aspiration and the plates were washed three times with RPMI medium under sterile conditions. Plates were blocked by incubating wells with 200 μl of RPMI medium containing 10% (vol/vol) fetal calf serum for at least 2 h at 37°C. A suspension containing 5 × 10^5^ lymphocytes isolated from popliteal and inguinal lymph nodes from treated mice was added to each well and cultured in 200 μl of RPMI containing 20 mM NaHCO3, 10 mM Hepes, 100 U/ml penicillin, 100 μg/ml streptomycin, 2 mM L-glutamine, 50 μM β-mercaptoethanol, 5 mM sodium pyruvate, 100 μM of non-essential amino acids solution and 2% normal mice serum and maintained for 72 h in the presence of *L. (L.) amazonensis* amastigote extract (corresponding to 1 × 10^7^ amastigotes/well). The lymphocytes were incubated for 72 h at 37°C in an atmosphere containing 5% CO_2_. After incubation, cultured cells were removed from the plates by washing two times with distilled water and three times with PBS-Tween. Each well received 100 μl of biotinylated anti-mouse IFN-γ diluted in PBS-Tween to a final concentration of 2 μg/ml. Plates were incubated for 2 h at room temperature, unbound antibodies were removed by washing the plates at least six times with PBS-Tween and 250 μg/ml of peroxidase-labeled streptavidin (HRP) was added. Plates were incubated for 1 h at room temperature and then washed three to five times with PBS-Tween and three times with PBS. The reactions on the plates were developed by adding 100 μl of freshly prepared AEC substrate solution (0.33 mg/ml 3-amino-9-ethylcarbazole, 3% N,N-dimethylformamide, 0,015% hydrogen peroxide in 0.1 M sodium acetate buffer, pH 5,0). After incubation at room temperature for 15 min, the reaction was stopped by discarding the substrate solution and rinsing the plates with water. Plates were dried at room temperature, and spots were counted with the aid of a stereomicroscope (Nikon).

Detection of active TGF-β was carried out in the supernatants of foot lesions from treated BALB/c and C57BL/6 mice. The animals were euthanized, and after homogenization of excised lesions in PBS, supernatants were collected, cleared by centrifugation, and assayed for TGF-β by using a double-sandwich ELISA assay according to the manufacturer instructions (eBioscience, Inc., San Diego, CA, USA). Supernatant concentrations higher than the minimal values obtained from the TGF-β standard were considered to be positive.

### Statistical Analysis

One-way ANOVA and Student’s *t*-test were used to determine the significant differences between groups by use of GraphPad Prism (version 5.0) and *P* values smaller than 0.05 (*P* < 0.05) were considered significant.

## Results

### Association with the Adjuvant *P. acnes* Increased the Leishmanicidal Effect of DPPE 1.2 in BALB/c and C57BL/6 Mice Infected with *L. (L.) amazonensis*

Previous data showed that the treatment of *L. (L.) amazonensis*-infected BALB/c mice with DPPE 1.2 was followed by increase of TCD4^+^ and TCD8^+^ lymphocyte populations (data not shown). These results and previous evidence that *P. acnes* exerts important immunomodulatory effects led us to use *P. acnes* as an adjuvant for the treatment with DPPE 1.2 in order to increase both the immune responses and the leishmanicidal activity of this palladacycle complex in BALB/c mice. In addition, a strain less susceptible to *L. (L.) amazonensis* infection, C57BL/6, was also used for the treatment with DPPE 1.2 plus *P. acnes*. Furthermore, as a positive control, both mouse strains infected with *L. (L.) amazonensis* were treated with Glucantime, the drug of choice for the treatment of leishmaniasis.

**FIGURE 1:**
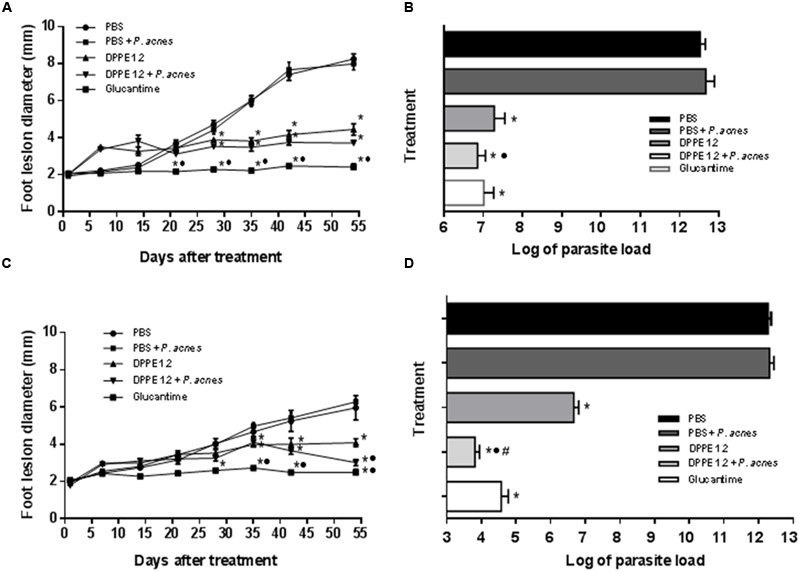
**Effect of DPPE 1.2 alone or associated with *P. acnes* on BALB/c (A,B)** and C57BL/6 mice **(C,D)** infected with *L. (L.) amazonensis*. **(A,C)** Development of foot lesions in treated mice. The treatment was started 15 days after infection and continued for 7 weeks. Data points represent the average measurements for five groups of 12 mice each. **(B,D)** Parasite load in foot lesions evaluated by number of parasites recovered by limiting dilution from treated *L. (L.) amazonensis*-infected mice. Parasites were quantified 15 days after interruption of treatment. Data are representative of three independent experiments. ^*^P < 0.05 compared to untreated animals; ^•^*P* < 0.01 compared to animals treated with DPPE 1.2 alone; ^#^*P* < 0.01 compared to animals treated with Glucantime.

**Figures 1A,C** show that starting from 28 days of treatment the animals which received either DPPE 1.2 or DPPE 1.2 + *P. acnes* showed a significant decrease of foot lesion size compared to controls. Animals treated with Glucantime also exhibited significantly smaller foot lesions compared to untreated control, as well as to animals treated with either DPPE 1.2 or DPPE 1.2 + *P. acnes*. Furthermore, C57BL/6 mice treated with DPPE 1.2 + *P. acnes* showed a significant reduction of foot lesion size compared to that observed in animals treated with DPPE 1.2 alone.

**FIGURE 2:**
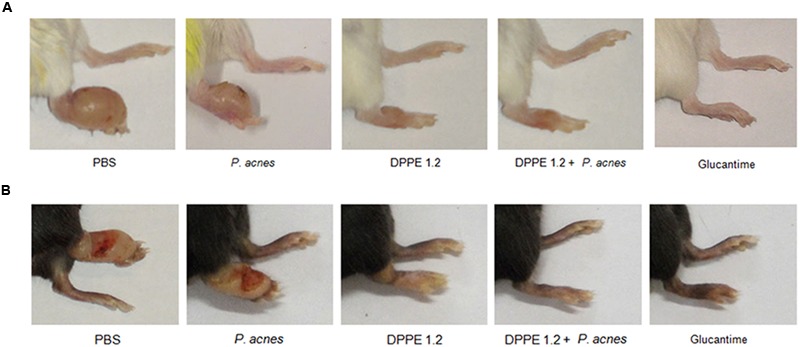
**Macroscopic evaluation of lesions in controls and treated mice.** *L. (L.) amazonensis-*infected BALB/c **(A)** and C57BL/6 **(B)** mice treated with either DPPE 1.2 alone, DPPE 1.2 associated with *P. acnes* or Glucantime 15 days after the end of treatment.

Parasite loads are shown in **Tables 1, 2** and **Figures 1B,D**. As can be observed, BALB/c and C57BL/6 mice treated with either DPPE 1.2, DPPE 1.2 + *P. acnes* or Glucantime displayed a significant reduction of parasite load compared to untreated animals. Among the three groups, parasite loads were significantly lower in both strains treated with DPPE 1.2 plus *P. acnes* compared to those estimated in animals treated with DPPE 1.2 alone. Furthermore, the parasite load of C57BL/6 mice treated with DPPE 1.2 plus *P. acnes* was also lower than that exhibited by animals treated with Glucantime (**Figure 1D**). **Figure 2** illustrates the macroscopic features of foot lesions in untreated mice or in mice treated with either DPPE 1.2 alone or associated with *P. acnes* or Glucantime 15 days after the end of treatment.

**Table 1:** Parasite load of *L. (L.) amazonensis*-infected BALB/c mice 15 days after interruption of treatment with DPPE 1.2 alone or associated with *P. acnes*.

Group	Parasite load
PBS	33.5 × 10^11^
PBS + *P. acnes*	46 × 10^11^
DPPE 1.2	1.92 × 10^7^
DPPE 1.2 + *P. acnes*	7.2 × 10^6^
Glucantime	1.03 × 10^7^

**Table 2:** Parasite load of *L. (L.) amazonensis*-infected C57BL/6 mice 15 days after interruption of treatment with DPPE 1.2 alone or associated with *P. acnes*.

Group	Parasite load
PBS	18.26 × 10^11^
PBS + *P. acnes*	20.55 × 10^11^
DPPE 1.2	4.66 × 10^6^
DPPE 1.2 + *P. acnes*	6.56 × 10^3^
Glucantime	3.74 × 10^4^

**FIGURE 3:**
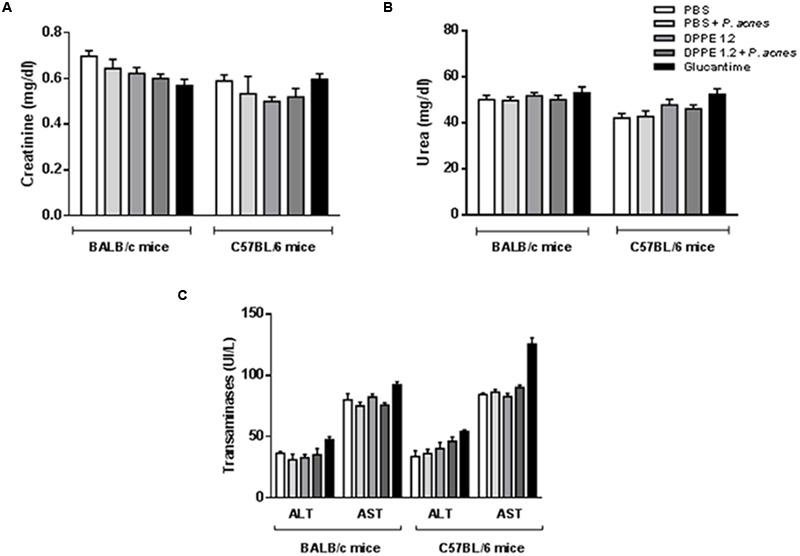
**Evaluation of toxicity in *L. (L.) amazonensis*-infected BALB/c and C57BL/6 mice after treatment with DPPE 1.2 alone or associated with *P. acnes*.** Serum concentrations of creatinine **(A)**, urea **(B)** and transaminases **(C)** in *L. (L.) amazonensis*-infected mice 15 days after the end of treatment with either DPPE 1.2, DPPE 1.2 plus *P. acnes* or Glucantime. The reference values are: creatinine: 0.3–1.0 mg/dl; urea: 34–58 mg/dl; transaminases: ALT: 53–202 UI/L, AST: 28–107 UI/L.

The hepato and nephrotoxicity of DPPE 1.2, DPPE 1.2 plus *P. acnes* and Glucantime was evaluated by determination of serum levels of transaminases, urea, and creatinine from treated mice. No statistically significant alterations were detected between groups (**Figure 3**).

### Increase of TCD4^+^ and CD8^+^ Lymphocytes in *L. (L.) amazonensis*-Infected BALB/c and C57BL/6 Mice after Treatment with DPPE 1.2 Associated with *P. acnes*

**FIGURE 4:**
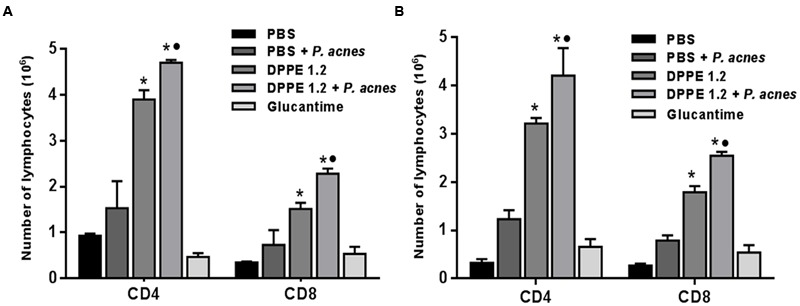
**Absolute number of T lymphocyte populations in treated mice.** *L. (L.) amazonensis*-infected BALB/c **(A)** and C57BL/6 **(B)** mice were treated with either DPPE 1.2 alone or associated with *P. acnes* or Glucantime. Lymphocytes were isolated from popliteal and inguinal lymph nodes, stained with monoclonal antibodies anti-CD3, anti-CD4 and anti-CD8 and analyzed by FACS. *^∗^P <* 0.01 compared to control that received PBS; ^•^*P <* 0.05 compared to group that received DPPE 1.2 alone.

The analysis of T lymphocyte expression by FACS was performed in the interval between the second and third administration of *P. acnes* in both mouse strains and until this time the animals had received either 1.92 mg/kg of DPPE 1.2 alone or associated to 200 μg of *P. acnes* or 360 mg/kg of Glucantime. There was a significant increase of TCD4^+^ and TCD8^+^ lymphocytes in BALB/c and C57BL/6 mice treated either with DPPE 1.2 or DPPE 1.2 + *P. acnes* compared to control groups (**Figure 4** and Supplementary Figure 1). Both strains treated with DPPE 1.2 plus *P. acnes* exhibited significantly higher number of TCD4^+^ and TCD8^+^ compared to those treated with DPPE 1.2 alone. No statistical differences in T lymphocyte expression was observed in animals treated with Glucantime compared to controls that received either PBS or PBS + *P. acnes*. The number of TCD4^+^ and TCD8^+^ lymphocytes was not significantly different between BALB/c and C57BL/6 mice treated with either DPPE 1.2 alone or associated to *P. acnes*.

Non-infected BALB/c and C57BL/6 mice treated with 1.92 mg/kg of DPPE 1.2 alone or associated to 200 μg of *P. acnes* did not show increase of TCD4^+^ and TCD8^+^ lymphocytes (Supplementary Figure 2).

### Treatment with DPPE 1.2 Associated with *P. acnes* Resulted in Increase of IFN-γ and Reduction of TGF-β Secretion in BALB/c and C57BL/6 Mice Infected with *L. (L.) amazonensis*

**FIGURE 5:**
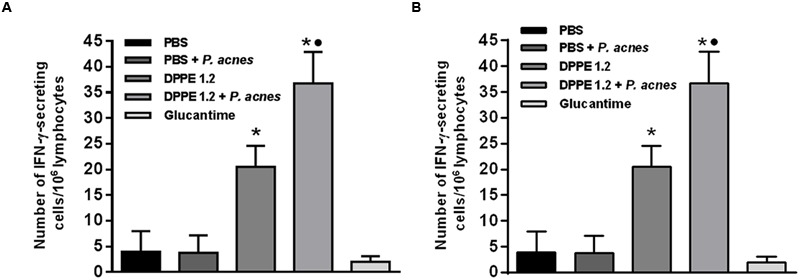
**Frequency of IFN-γ-secreting lymphocytes in treated mice.** BALB/c **(A)** and C57BL/6 **(B)** mice infected with *L. (L.) amazonensis* were treated with either DPPE 1.2 alone or associated with *P. acnes* or Glucantime. Lymphocytes were isolated from popliteal and inguinal lymph nodes and after 96 h of stimulation *in vitro* with extract of *L. (L.) amazonensis* amastigotes were detected by ELISPOT technique. ^∗^*P* < 0.05 compared to control that received PBS; ^•^*P <* 0.05 compared to group that received DPPE 1.2 alone.

At the same period of treatment lymphocytes secreting IFN-γ and secretion of TGF-β from treated mice were evaluated. **Figure 5** shows that higher frequency of IFN-γ-secreting lymphocytes was found in BALB/c and C57BL/6 mice treated with either DPPE 1.2 alone or associated with *P. acnes* compared to those that received PBS. The increase of IFN-γ-secreting lymphocytes was significantly higher in animals treated with DPPE 1.2 plus *P. acnes* compared to those treated with DPPE 1.2 alone, while no difference in IFN-γ production was observed between the two mouse strains.

**FIGURE 6:**
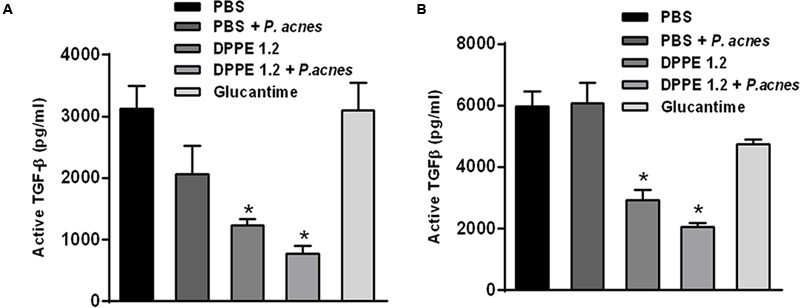
**Evaluation of TGF-β in treated mice.** Levels of active TGF-β were determined by ELISA assay in supernatants of foot lesions from *L. (L.) amazonensis* infected BALB/c **(A)** and C57BL/6 mice **(B)** treated with either DPPE 1.2 alone or associated with *P. acnes* or Glucantime. ^∗^*P* < 0.01 compared to control groups.

Data on TGF-β dosages are shown in **Figure 6**. High levels of active TGF-β were detected in the foot lesions from mice that received either PBS, *P. acnes* or Glucantime, in contrast to those treated with either DPPE 1.2 alone or associated with *P. acnes* that displayed a significant reduction of TGF-β production. Although both mouse strains treated with DPPE 1.2 plus *P. acnes* displayed lower concentrations of TGF-β than those treated with DPPE 1.2 alone, this difference was not significant. It was also observed that the concentration of active TGF-β in lesions of BALB/c mice was about twofold lower than that found in C57BL/6. However, the reduction of TGF-β in treated animals was not significantly different between the two strains.

## Discussion

The present study compared the effect of DPPE 1.2 associated with *P. acnes* on *L. (L.) amazonensis* infection in BALB/c and C57BL/6 mice. All animals from both strains treated with DPPE 1.2 displayed a significant reduction of parasite load and this reduction was significantly higher in mice treated with DPPE 1.2 plus *P. acnes*. The treatment with DPPE 1.2 resulted in a twofold higher reduction of parasite load in C57BL/6 mice compared to that observed in BALB/c strain, while this difference was of 600-fold between the two strains treated with DPPE 1.2 plus *P. acnes*. These results reflect the different susceptibility to *L. (L.) amazonensis* of these strains, since BALB/c mice develop an anergic form of diffuse cutaneous leishmaniasis characterized by metastasizing and non-self-healing foot lesions harboring a high number of amastigotes (Andrade et al., 1984). On the other hand, infection by *L. (L.) amazonensis* in the C57BL/6 strain generates lesions which tend to chronicity (Calabrese and Costa, 1992). Therefore, the higher difference observed between the two mouse strains treated with DPPE 1.2 plus *P. acnes* probably is due to intrinsic characteristics of the C57BL/6 strain that resulted in a more responsiveness to the stimulus induced by the adjuvant and in a higher protection in these animals. However, despite of the significant increase of CD4^+^ and CD8^+^ T lymphocytes in animals treated with DPPE 1.2 plus *P. acnes* compared to that observed in mice treated with DPPE 1.2 alone, no differences were observed in the number of these T lymphocyte populations between the two strains after treatment with DPPE 1.2 plus *P. acnes*.

The higher frequency of lymphocytes producing IFN-γ and the increase of T lymphocytes in mice treated with either DPPE 1.2 or DPPE 1.2 plus *P. acnes* indicate the involvement of CD8^+^ and CD4^+^ Th1 cells in the production of this lymphokine in these groups. The participation of TCD4^+^ Th1 lymphocytes producing IFN-γ has been demonstrated in mice protected against *L. (L.) amazonensis* infection (Coelho et al., 2003; Pinto et al., 2004). Furthermore, the involvement of CD8^+^ T lymphocytes producing perforin and IFN-γ was also demonstrated in BALB/c mice immunized with *Leishmania* antigen and protected against a challenge with *L. (L.) amazonensis* (Colmenares et al., 2003). It is also important to emphasize that the significant increase of CD4^+^ and CD8^+^ T lymphocytes, as well as the IFN-γ production in mice treated with DPPE 1.2 plus *P. acnes* compared to those treated with DPPE 1.2 alone are in accordance with previous data that showed that treatment with *P. acnes* elicits a type-1 (Th1) immune response involving IL-12 and IL-18 that induces IFN-γ release (Matsui et al., 1997). Administration of killed *P. acnes* as an adjuvant increased the resistance to infection by *T. cruzi* (Mussalem et al., 2006) and in leishmaniasis this adjuvant was used in murine vaccination with native and recombinant antigens resulting in protective immunity mediated by CD4^+^ Th1 lymphocytes (Pinto et al., 2000; Ferreira et al., 2008). More recently, *P. acnes* was also used in association to a recombinant cysteine proteinase from *L. (L.) infantum chagasi* for treatment of canine visceral leishmaniasis (Ferreira et al., 2014).

Data on IFN-γ production suggest the involvement of this cytokine in parasite destruction of mice treated with either DPPE 1.2 or DPPE 1.2 plus *P. acnes*. However, it is possible to assign to cytotoxic CD8^+^ lymphocytes a more relevant role in parasite load decrease in treated mice as recent data showed that the *L. (L.) amazonensis* strain used in the present study is unresponsive to nitric oxide secreted by macrophages activated by IFN-γ (Carmo et al., 2010). This hypothesis is supported by previous data which showed an increase of CD8^+^ expression parallel to their cytotoxic activity on *L. (L.) amazonensis*-infected macrophages from BALB/c mice immunized with a recombinant cysteine proteinase from *L. (L.) amazonensis* and partially protected against homologous infection (Fedeli et al., 2010). Furthermore, the relevant role of cytotoxic TCD8^+^ lymphocytes in *L. (L.) amazonensis* infection was demonstrated by other authors (Alves et al., 2004; Pereira and Alves, 2008; Pereira et al., 2011; Souza-Silva et al., 2014).

TGF-β was demonstrated to exacerbate infection by cutaneous and visceralizing *Leishmania* species (Barral-Netto et al., 1992; Pinheiro et al., 2005). The present data corroborate these results since low levels of TGF-β were detected in foot lesions from mice treated with either DPPE 1.2 or DPPE 1.2 plus *P. acnes* with a concomitant reduction of parasite load, increase of CD4^+^ and CD8^+^ T lymphocytes and significant secretion of IFN-γ. Secretion of IFN-γ was not different between BALB/c and C57BL/6 mice treated with either DPPE 1.2 or DPPE 1.2 plus *P. acnes*, while the production of TGF-β was about twofold higher in C57BL/6 strain. However, the reduction of active TGF-β levels in animals treated with either DPPE 1.2 or DPPE 1.2 plus *P. acnes* was similar between the two strains.

The treatment of *L. (L.) amazonensis*-infected BALB/c mice with either DPPE 1.2 alone or Glucantime resulted in a similar reduction of parasite load. On the other hand, in C57BL/6 strain the treatment with Glucantime resulted in a significantly higher reduction of parasite load than that observed in animals treated with DPPE 1.2 alone, whereas this reduction was lower when compared to that evaluated in mice treated with DPPE 1.2 plus *P. acnes*. These findings strengthen that C57BL/6 is more responsive to the stimulus induced by *P. acnes*, as discussed above. It is worth noting that although the reduction of parasite load in BALB/c mice treated with DPPE 1.2 was similar to that obtained with Glucantime, this antimonial compound was used in 200-fold higher concentration. The leishmanicidal mechanism of Glucantime has not been clearly elucidated, but several reports show that direct and indirect mechanisms are involved in *Leishmania* destruction by this antimonial and the host immune system has also been implicated in its leishmanicidal activity (Muniz-Junqueira and de Paula-Coelho, 2008). Our findings showed that in both mouse strains the treatment with Glucantime did not increase the number of CD4^+^ T and CD8^+^ T lymphocytes. Moreover, IFN-γ and TGF-β secretion in foot lesions of animals treated with Glucantime was not different than that observed in non-treated controls, indicating that in both mouse strains the leishmanicidal activity of Glucantime is not mediated by activation of cellular immune response against *L. (L.) amazonensis*.

Interestingly, our data also showed that is crucial the presence of parasites for the immunomodulatory effect of DPPE 1.2, since the administration of this palladacycle complex to non-infected mice did not result in activation of the immune system. This is certainly related to the leishmanicidal mechanism of DPPE 1.2 that needs to be further explored.

## Conclusion

The present study strengthened our previous results on activity of DPPE 1.2 on *L. (L.) amazonensis* and showed that *in vivo* treatment with this palladacycle complex led to the stimulation of cellular immune responses mediated by both CD4^+^ and CD8^+^ T lymphocytes in treated mice. Furthermore, a significantly higher reduction of foot lesion size and parasite burden followed by a higher increase of CD4^+^ and CD8^+^ T lymphocytes could be observed in both strains treated with DPPE 1.2 plus *P. acnes* compared to that found in animals treated with DPPE 1.2 alone. These findings open perspectives to explore the potential of DPPE 1.2 associated with *P. acnes* as an additional option for the chemotherapy of leishmaniasis.

## Author Contributions

CP, DdS, PM, DT, and SK: Designed and performed experiments, analyzed and interpreted the data. DG: Design, synthesis, and analysis of the compound. IL-M: Analyzed and interpreted the data and contributed to reviewing of manuscript. CB: Conceived the work, contributed to interpretation of data, wrote and reviewed the manuscript.

## Conflict of Interest Statement

The authors declare that the research was conducted in the absence of any commercial or financial relationships that could be construed as a potential conflict of interest.

## Footnotes

1 http://www.cobea.org.br/
