# Supplementary figures and images for: Treatment of Leishmania (Leishmania) Amazonensis-Infected Mice with a Combination of a Palladacycle Complex and Heat-Killed Propionibacterium acnes Triggers Protective Cellular Immune Responses
Source: Front Microbiol. 2017 Mar 6;8:333. doi: 10.3389/fmicb.2017.00333 (PMC5337482; doi:10.3389/fmicb.2017.00333)

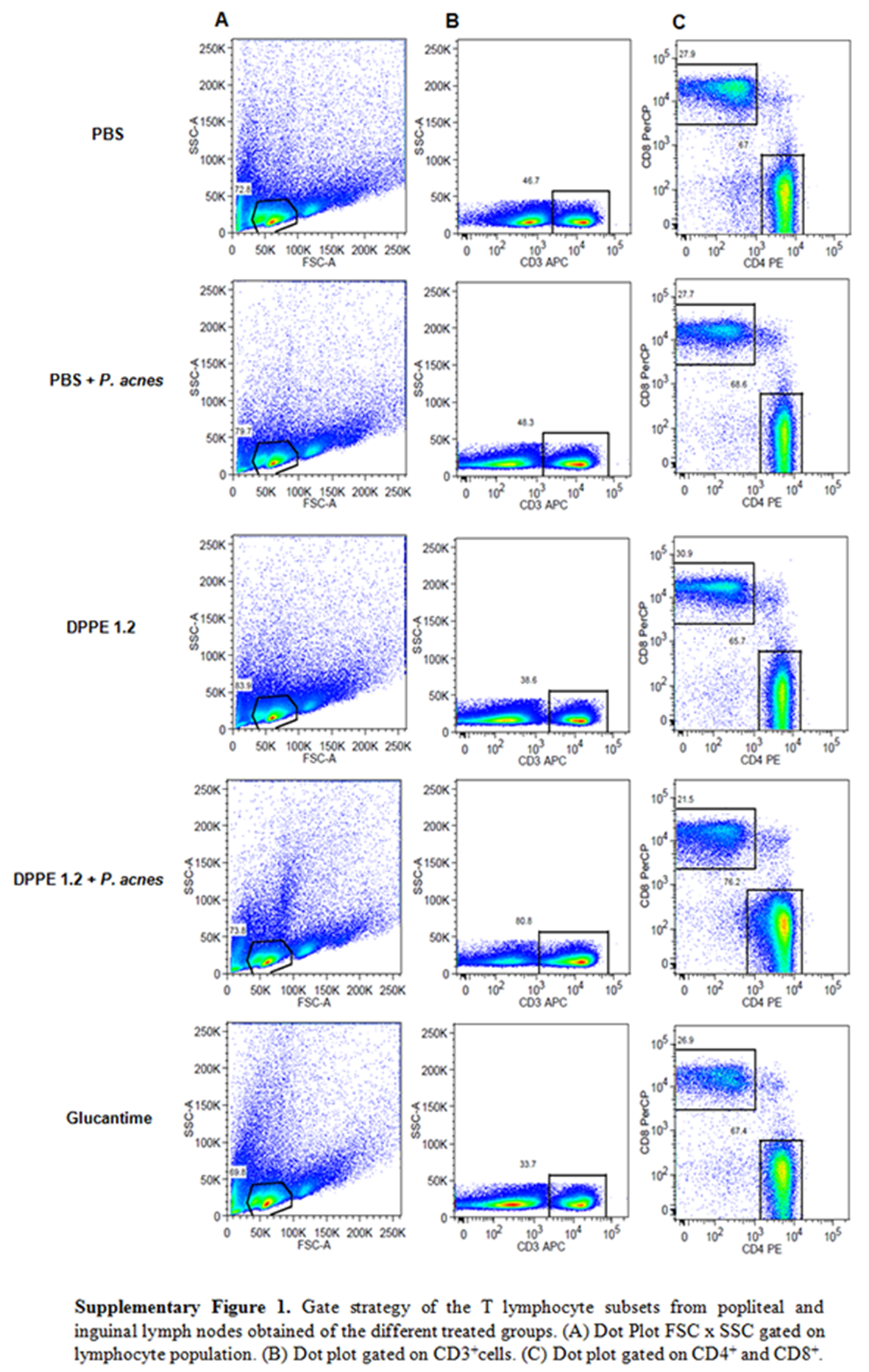

Supplement: Supplementary file 1 [file Image_1.tif]

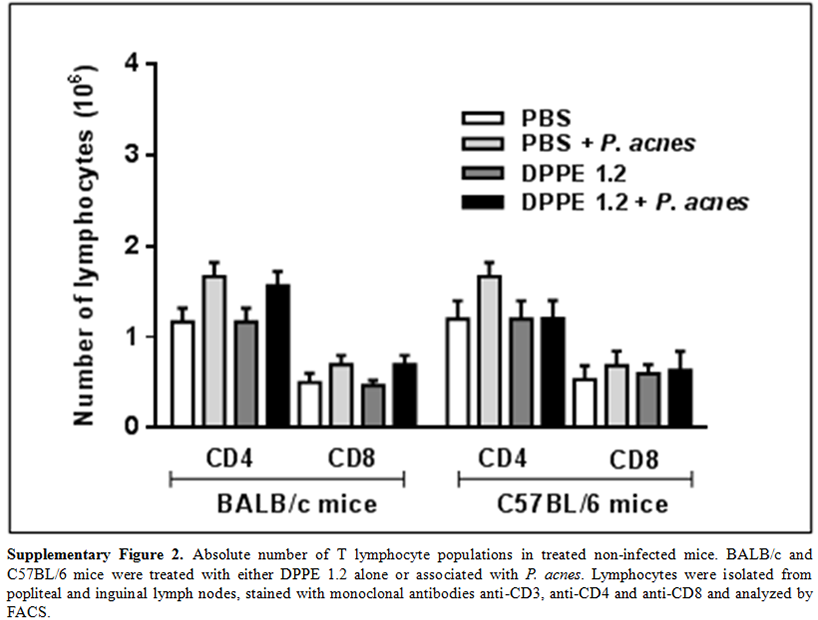

Supplement: Supplementary file 2 [file Image_2.tif]
